# Merging and Fractionation of Muscle Synergy Indicate the Recovery Process in Patients with Hemiplegia: The First Study of Patients after Subacute Stroke

**DOI:** 10.1155/2016/5282957

**Published:** 2016-12-19

**Authors:** Yu Hashiguchi, Koji Ohata, Ryosuke Kitatani, Natsuki Yamakami, Kaoru Sakuma, Sayuri Osako, Yumi Aga, Aki Watanabe, Shigehito Yamada

**Affiliations:** ^1^Department of Physical Therapy, Faculty of Health Science, Gumma Paz College, Gunma, Japan; ^2^Department of Physical Therapy, Human Health Sciences, Graduate School of Medicine, Kyoto University, Kyoto, Japan; ^3^Kansai Rehabilitation Hospital, Osaka, Japan; ^4^Fujita Health University Hospital, Aichi, Japan; ^5^Department of Rehabilitation Sciences, Kansai University of Welfare Science, Osaka, Japan; ^6^Kuretake Special Support School, Kyoto, Japan; ^7^Aijinkai Rehabilitation Hospital, Osaka, Japan; ^8^Oita Tobu Hospital, Oita, Japan

## Abstract

Loss of motor coordination is one of the main problems for patients after stroke. Muscle synergy is widely accepted as an indicator of motor coordination. Recently, the characteristics of muscle synergy were quantitatively evaluated using nonnegative matrix factorization (NNMF) with surface electromyography. Previous studies have identified that the number and structure of synergies were associated with motor function in patients after stroke. However, most of these studies had a cross-sectional design, and the changes in muscle synergy during recovery process are not clear. In present study, two consecutive measurements were conducted for subacute patients after stroke and the change of number and structure of muscle synergies during gait were determined using NNMF. Results showed that functional change did not rely on number of synergies in patients after subacute stroke. However, the extent of merging of the synergies was negatively associated with an increase in muscle strength and the range of angle at ankle joint. Our results suggest that the neural changes represented by NNMF were related to the longitudinal change of function and gait pattern and that the merging of synergy is an important marker in patients after subacute stroke.

## 1. Introduction

Motor dysfunction due to neural disorders is responsible for several complications in patients recovering from stroke [1]. In particular, gait disorders can affect the patient's ability to participate in daily activities [2]. Impairments resulting in gait disorder have been reported previously, such as muscle weakness [3], spasticity [4], and, most importantly, poor motor coordination [5].

Previous studies have investigated the problem of motor coordination using cocontraction between agonist and antagonist muscles in patients after stroke [5–7]. However, the effects of cocontraction on gait were not consistent in these studies. One study suggested that excessive cocontraction during gait may adversely affect the energy cost during gait [7], whereas another study reported that cocontraction is needed as an adaptive behavior for retaining stability during gait [5]. This inconsistency in evidence reflects the limitations of using cocontraction as an indicator of motor coordination during gait. Therefore, a more comprehensive and specific indicator is needed for motor coordination in patients after stroke.

In general, the brain needs to coordinate the degrees of freedom in the musculoskeletal system during movement [8], and muscle synergy is hypothesized to manage the problem with degrees of freedom [9]. Based on this hypothesis, the central nervous system controls muscle synergy, which comprehensively coordinates the activation of several muscles during movement. Recent studies have demonstrated that the number and structure of muscle synergies can be directly identified using nonnegative matrix factorization (NNMF) with surface electromyography (EMG) during gait and reaching tasks [10–12]. The physiological validity and robustness of this method have been demonstrated in previous studies [13, 14].

Using this method, it was reported that the number of synergies did not change in patients with spinal cord injury (SCI) [15]. However, the evidence is conflicting in poststroke patients, with one study demonstrating a similar number of synergies in poststroke patients and healthy adults [16] and another reporting a decreased number of synergies in patients after stroke [17]. These contradictory results may be caused by differences in the duration after stroke. The study that reported no changes in the number of synergies recruited patients after subacute stroke [16], whereas the study demonstrating a reduced number of synergies recruited patients after chronic stroke [17]. Furthermore, one previous study investigated the change in muscle synergy in patients after chronic stroke [18]. The study demonstrated that muscle synergies were fine-tuned and that the number of synergies was increased in some patients with improved motor function. However, the change in muscle synergies, including the number and structure of synergies, in patients after subacute stroke has not been clarified.

Of clinical importance is another previous study that demonstrated that muscle synergy calculated by NNMF was strongly associated with a dynamic response during movement [19]. For stroke patients, abnormal gait patterns were often represented by gait kinematics and kinetics. For example, abnormal gait kinematics were mostly represented at the knee joint or ankle joint [20], whereas the change in gait kinetics relating to gait function was shown at the ankle joint [21]. However, the longitudinal relationship between muscle synergy and gait dynamics in stroke patients is unknown.

Another study reported that the merging and fractionation of muscle synergies could explain the changes in the number of synergies in patients after stroke [22]. The degrees of merging and fractionation were individually associated with the characteristics of patients. Merging was related to impairment of the upper limbs, and fractionation was related to the duration after stroke. Thus, it is considered that merging and fractionation could be used as indicators of motor coordination in patients after stroke. However, the mechanism of how these changes occurred and whether they are related to the recovery of motor function remain unclear.

Furthermore, most results regarding muscle synergy were demonstrated by cross-sectional studies. However, longitudinal changes in muscle synergy are still unknown. We conducted two consecutive measurements and clarified the changes in the number and structure of muscle synergies in patients after subacute stroke and investigated the relationship between the change in muscle synergy and change in motor function or gait dynamics during the recovery process.

## 2. Materials and Methods

### 2.1. Participants

This study was conducted at the Yufuin Kosei Nenkin Hospital in Oita, Japan. Patients with the following inclusion criteria were recruited: (1) a single stroke within 6 months prior to the study; (2) ability to walk independently using an ankle-foot orthosis or T-cane; (3) no gait symptoms from Parkinson's or ataxia; (4) no pain during gait due to orthopedic disease; (5) no limitation of activity due to heart disease; and (6) no difficulty in understanding the experimental tasks due to cognitive problems. Thirteen patients who had experienced subacute stroke met the inclusion criteria and participated in this study (mean time elapsed after stroke: 66.8 ± 24.2 days). The patients' clinical characteristics are presented in Table 1. This study was approved by the Ethics Committee of Kyoto University Graduate School, Faculty of Medicine, and Yufuin Kosei Nenkin Hospital, and we obtained informed consent from all patients.

### 2.2. Experimental Protocol and EMG Recordings

Two measurements (first and second measurements) were performed at 1-month intervals. Between the first and second measurements, all patients participated in the inpatient rehabilitation program, which included gait training, balance training, and task-specific training for activities of daily living (ADL) for 60 minutes per day, five times per week. During each recording, gait measurements and clinical measurements were performed. For the gait measurement, two gait trials were performed by asking patients to walk a 10 m long walkway at a chosen speed with or without a cane. Muscle activity was recorded simultaneously with surface EMG (sEMG) using a Trigno Wireless System (Delsys Co., Boston, USA; sampling rate: 4000 Hz), which also recorded the data from a 3D accelerometer (ACC). The sEMG activity was recorded from the tibialis anterior (TA), lateral gastrocnemius (GS), soleus (SL), gluteus medius (GM), rectus femoris (RF), vastus medialis (VM), biceps femoris (BF), and semitendinosus (ST) muscles of the affected side, and another sensor was placed on the heel of the measured limb to record the ACC data.

The corrected sEMG data were bandpass-filtered (20–250 Hz), rectified, and then low-pass filtered (10 Hz). Each gait cycle was determined by ACC data and normalized to 200 data points. Furthermore, the amplitude was normalized to the peak activity recorded during five gait cycles. A factor analysis was performed with the normalized data (nEMG).

### 2.3. Muscle Synergy Extraction

For each subject, the nEMG data were separated into patterns of synergies and muscle weightings using an NNMF algorithm [12]. The nEMG data *m*(*t*) are represented by the following equation:

(1) $$\begin{array}{c} m\left(\;t\;\right)=\overset{n}{\underset{i-1}{\sum }}C_{i}\left(\;t\;\right)W_{i}. \end{array}$$

This algorithm could reveal synergies in the following two matrices: *C*_*i*_(*t*), which denotes the activation pattern of each synergy during five gait cycles (*n* ×* t* matrix;* n* = number of synergies,* t* = time point), and *W*_*i*_, which represents the weightings of the muscles involved in each synergy (*m* ×* n* matrix;* m* = eight muscles of the paretic leg). The NNMF algorithm was initialized with two random matrices of activation patterns and weightings. The nEMG data were reconstructed by iteratively updating the values of these matrices until they converged.

### 2.4. Determining the Number of Synergies for Each Subject and Group

The NNMF analyses were performed with the output restricted to one, two, three, four, or five synergies, with no a priori assumptions about the adequate number of synergies. The reconstructed EMG (rEMG) was calculated by performing matrix multiplication, with both matrices indicating the activation pattern and weightings of synergies; the sum of squared errors (nEMG-rEMG) was then calculated. The variability accounted for (VAF), which was the ratio of the sum of the squared error to the sum of the squared nEMG, was calculated to determine whether the minimum number of synergies corresponded with adequate rEMG. Because the threshold of VAF could potentially change the number of synergies, we used the threshold from a previous study [17]. We determined that additional synergies were not required if VAF including all muscles was ≥90%.

### 2.5. Merging and Fractionation Indices

**Figure 1:**
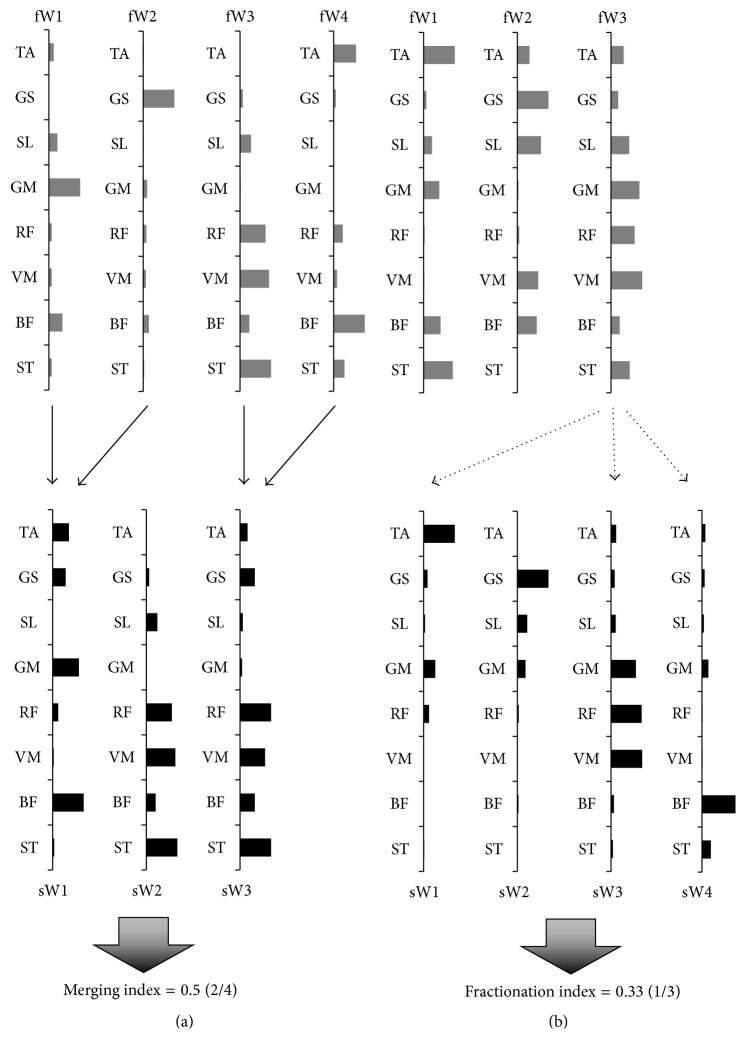
Merging and fractionation of the synergies. The figures show the merging (solid line) and fractionation (dotted line) of the synergies at the first (gray) and second measurements (black). (a) Merging of synergies recorded from a patient; the weighting of the sW2 and sW3 synergy was reconstructed by linearly combining two pairs of synergies (fW1 and fW2, fW3 and fW4) from the first measurement. (b) Fractionation of the synergies that were recorded; the weighting of the fW3 synergy at the first measurement was reconstructed by linearly combining three synergies (sW1, sW3, and sW4) from the second measurement.

The change in structure of muscle synergy was investigated using merging and fractionation indices calculated with a linear combination, as reported in a previous study [22]. The merging index was defined as the ratio of the frequency of merging of the synergy to the total number of synergies in the first measurement, whereas the fractionation index was defined as the ratio of frequency of fractionation of the synergy to the total number of synergies in the first measurement (Figure 1).

### 2.6. Clinical Measures

The gait speed of each patient was measured using a stopwatch. The Timed Up and Go test (TUG) and the Short-Form Berg Balance Scale (SFBBS) were used to assess the function of dynamic or static balance for each patient. The Barthel index (BI) was measured as a functional outcome of ADL. Furthermore, the muscle strength (N·m) of five muscles (hip flexor, knee extensor, knee flexor, ankle dorsiflexor, and ankle plantar flexor) was measured using a hand-held dynamometer (*μ*-tas F-1; ANIMA Corp., Tokyo, Japan) and normalized by weight (N·m/kg). The sum of the five muscle strengths represented the parameter for all the muscles. Furthermore, the change in the parameters of motor function (Δ speed, Δ TUG, Δ SFBBS, Δ BI, and Δ strength) was represented by the ratio of change among trials involving the value of the first measurement.

### 2.7. Kinematical Measures

A follow-up gait measurement was also performed in the same motion analysis laboratory. The laboratory had a 3D motion analysis system (T-10; Vicon Motion System Ltd., Oxford, UK) with eight cameras and a sampling frequency of 100 Hz. Reflective markers were attached to the body according to the Vicon Plug-in-Gait (PiG) marker placement protocol (full body). Data were processed using PiG software, which uses a Woltring filter, and joint kinematics were generated using inverse dynamics analysis within Nexus version 1.7 software (Vicon Motion System Ltd.). The data recorded by 3D motion analysis were time-normalized to 100% gait cycle (GC). The parameters of gait kinematics at the hip, knee, and ankle joints were detected as the peak of the joint angle during the gait cycle, as shown in Table 2. Furthermore, the changes in gait kinematics between measurements were calculated as the difference or ratio of change in peak or angle range, respectively.

### 2.8. Statistical Analyses

First, interclass correlation coefficients (ICC_(1,1)_) were calculated between the two trials of the first measurements to investigate the test-retest reliability of the number of synergies indicated by NNMF. Second, the paired* t*-test and Wilcoxon signed-rank test were used to examine the differences in clinical parameters, gait kinematics, and the number of synergies between the two measurements. Furthermore, the relationship between the merging index or fractionation index and the change in clinical parameters or gait kinematics was investigated using multiple linear regression with stepwise procedures and using the change in clinical parameters and gait kinematics at three joints as explanatory variables and the merging and fractionation indices as target variables.

## 3. Results

### 3.1. Validity of the Number of Synergies

Table 3 shows the changes in the number of synergies, merging and fractionation indices, and the motor function at the first measurement. Regarding the number of synergies, the results showed high test-retest reliability (ICC = 0.81, almost perfect).

### 3.2. Change in the Motor Function and Number of Muscle Synergies

Furthermore, the results showed that gait speed and muscle strength had significantly improved (*p* < 0.01 and *p* < 0.05, resp.). Other parameters including BI, TUG, and BBS also significantly improved. However, no consistent changes in the number of synergies between the first and second measurements were found (*p* = 0.73).

In addition, the kinematics did not show a significant change between two measurements. The peaks of flexion at the hip (hip F1) and knee (knee F1, F2) tended to increase after a month; however, the other peak angles at the three joints did not show a consistent change between two measurements. Furthermore, the ranges of hip and knee joints had increased since the first measurement; however, the ankle joint range did not show a consistent change.

### 3.3. Relationship between the Changes in Muscle Synergy and Motor Function or Gait Kinematics

Merging of synergy was observed in eight patients (61.5%) after stroke, whereas fractionation was found in 10 patients (76.9%) after stroke. The merging index was associated with the change of muscle strength and range of the ankle joint with a significant coefficient of determination (Table 4). However, the fractionation index was significantly related to only the improvement in BI. Changes in gait speed, SFBBS, and TUG were not significantly associated with the merging or fractionation indices.

## 4. Discussion

Our present study clarified the longitudinal change in muscle synergy calculated using NNMF for patients after subacute stroke. The high test-retest reliability was confirmed by the number of synergies calculated by NNMF. Using this method, a consistent change in the number of synergies was not found at monthly measurements, even though patients had significantly improved gait speed. However, merging of synergy was found in 61.5% of patients and fractionation of synergy was found in 76.9% of patients in this study. Furthermore, the extent of merging and fractionation depended on motor function and gait dynamics.

A previous study showed a change in timing and composition of muscle synergy in chronic stroke patients [18]. The results showed that the fine-tuning of muscle synergy and the increase in the number of synergies were associated with improvement in motor function. However, in this study, a consistent increase or decrease of the number of synergies was not found in subacute stroke patients with improved motor function. The results reflected that the neural networks relating to the number of synergies in subacute stroke patients did not change homogeneously.

**Figure 2:**
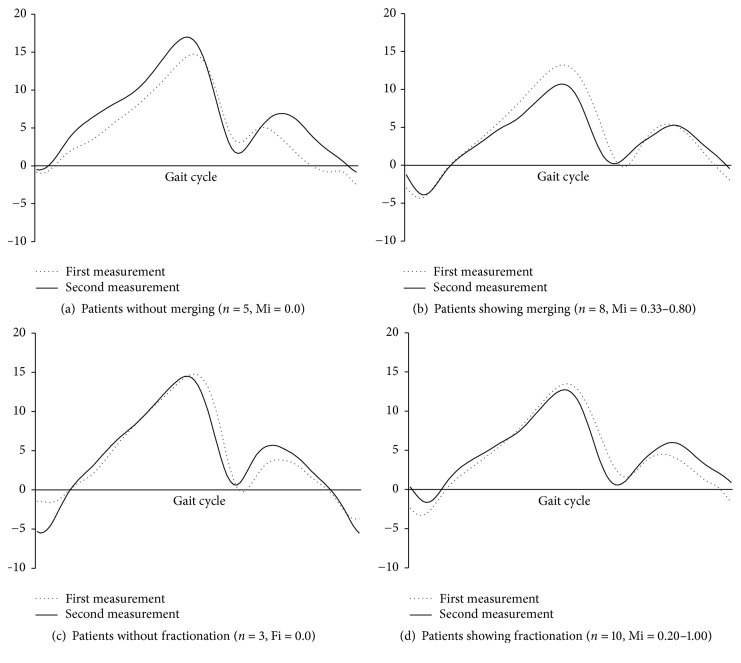
The change of ankle joint angle. The group with merging (b) showed limitation in the range of the ankle joint angle. The patients who did not show merging (a) had the same joint angle range. The group without or with fractionation (c and d) did not show the consistent change of gait kinematics.

In a previous cross-sectional study, severe stroke patients showed higher merging index and lower motor function as estimated by the Fugl-Meyer scale [22]. As a longitudinal change in present study, the present results suggest that higher merging index was associated with poor improvement of outcome, such as muscle strength and gait kinematics. Specifically, the patients with merging of synergy had poor improvement in muscle strength and restriction of ankle joint range at monthly measurements (Figure 2). The merging of synergy indicated by NNMF is thought to represent synchronization of the neural network. Therefore, it is considered that the merging of synergy may represent the compensative neural change to achieve a dynamic response after improvement of gait.

The fractionation index was associated with duration after stroke in a previous study [22]. In the present study, an association between the fractionation and duration after stroke was not found because the patients were inpatients. However, the fractionation index was related to the improvement in ADL. This suggested that the fractionation of synergy was influenced by the complexity of several movements in ADL during the recovery process. However, the mechanism of fractionation of synergy is not clear. Future studies are needed to investigate the background of fractionation of synergy.

The present study had some limitations. First, our sample size was small for the convenience of sampling, resulting in a small range of variability of motor function in the subjects. Future studies should include patients with more severe symptoms to allow a better understanding of the characteristics of synergy behavior. Other limitations were the small number of gait cycles assessed and use of a cane by some patients. These methodologies could affect the extraction of muscle synergies. However, a previous study also used a small number of gait cycles (10 gait cycles) for patients with SCI who were unable to walk long distances. Thus, a larger number of gait cycles and gait measurements without a cane are required to accurately investigate the synergy during gait. Furthermore our results showed the neural change during the short period, in which the patients significantly improved motor function. These results could not demonstrate the full recovery process in patients after stroke. Therefore, future studies should also measure the muscle synergy and motor function at more time points to clarify the long-term changes in muscle synergy in patients after stroke.

## 5. Conclusion

The results of this study showed that the number of synergies did not consistently change with the recovery of motor function in subacute stroke patients. The merging and fractionation of the synergies during gait occurred depending on motor function. The merging of synergy was especially related to the unchanged muscle strength and abnormal gait pattern. The results of the present study suggest that NNMF can be used to clarify the characteristics of motor coordination in stroke patients and that the merging of synergies is thought to be an important marker of poor motor coordination.

## Figures and Tables

**Table 1:** General characteristics of stroke patients.

	*N* = 13
Sex (M/F)	10/3
Age (years) [range]	58.8 ± 13.2 [30–82]
Height (cm)	160.2 ± 7.3
Weight (kg)	65.4 ± 11.7
Brunnstrom stage (V/VI)	(11/2)
Duration after stroke (day) [range]	66.8 ± 24.2 [38–118]
Barthel index [range]	86.5 ± 9.9 [65–95]
Gait speed (m/sec) [range]	0.54 ± 0.24 [0.50–1.38]

M: male; F: female.

Data are expressed as mean ± SD and range for stroke patients.

**Table 2:** Determined peak position during gait cycle at three joints.

Parameters	Joint	Peak motion	Range (% gait cycle)	
			From	To
HF1	Hip	Flexion	0	20
HE		Extension	0	100
HF2		Flexion	90	100
KF1	Knee	Flexion	0	20
KE		Extension	20	50
KF2		Flexion	50	100
AP1	Ankle	Planter flexion	0	20
AD		Dorsiflexion	0	100
AP2		Planter flexion	50	70

**Table 3:** Affected side, synergy information, and clinical status.

Patients	Affected side	Duration after stroke (days)	Synergy number at first measurement	Synergy number at second measurement	Merging index	Fractionation index	Gait speed	Barthel index	BRSs
1	L	65	2	2	0.00	0.50	0.50	85	4
2	L	69	2	2	0.00	0.00	0.54	95	5
3	L	46	2	3	0.00	1.00	0.86	90	5
4	R	45	3	3	0.00	0.000	0.51	85	5
5	R	62	3	4	0.00	1.00	0.85	95	5
6	L	46	3	4	0.33	1.00	0.62	65	5
7	L	115	3	3	0.33	0.33	0.99	90	6
8	L	80	3	3	0.33	0.00	0.80	95	5
9	R	74	3	4	0.67	0.33	1.10	90	5
10	L	118	4	3	0.50	0.25	1.38	95	6
11	L	56	5	5	0.80	0.40	0.73	90	5
12	L	55	5	3	0.60	0.40	0.69	85	5
13	R	38	5	3	0.60	0.20	0.93	65	5

L: left; R: right.

**(a):** 

Merging index (*y*)	Model *R* ^2^	Predictors (*x*)	*β*	95% CI	*p*
Model 1: strength	0.427	Intercept			<0.01
		Strength	−0.651	−1.47, −0.19	<0.05
Model 2: strength/range of ankle	0.647	Intercept			<0.01
		Strength	−0.558	−1.26, −0.17	<0.05
		Range of ankle	−0.481	−1.16, −0.07	<0.05

**(b):** 

Fractionation index (*y*)	Model *R* ^2^	Predictor (*x*)	*β*	95% CI	*p*
Model 1: BI	0.333	Intercept			<0.05
		BI	0.577	0.15, 4.84	<0.05
